# The interplay between gut microbiota, short-chain fatty acids, and implications for host health and disease

**DOI:** 10.1080/19490976.2024.2393270

**Published:** 2024-09-16

**Authors:** Kallie E. Hays, Jacob M. Pfaffinger, Rebecca Ryznar

**Affiliations:** a Doctor of Osteopathic Medicine Program, Rocky Vista University College of Osteopathic Medicine, Englewood, CO, USA; b Department of Biomedical Sciences, Rocky Vista University College of Osteopathic Medicine, Englewood, CO, USA

**Keywords:** Short-chain fatty acids (SCFAs), gut microbiome, bacterial metabolites, SCFA production pathways, butyrate, acetate, propionate, butyrate-producing bacteria, antibiotics, antibiotic therapy, gram-positive antibiotics, *Roseburia*

## Abstract

Short-chain fatty acids (SCFAs) – acetate, propionate, and butyrate – are important bacterial fermentation metabolites regulating many important aspects of human physiology. Decreases in the concentrations of any or multiple SCFAs are associated with various detrimental effects to the host. Previous research has broadly focused on gut microbiome produced SCFAs as a group, with minimal distinction between acetate, propionate, and butyrate independently, each with significantly different host effects. In this review, we comprehensively delineate the roles of these SCFAs with emphasis on receptor affinity, signaling pathway involvement, and net host physiologic effects. Butyrate is highlighted due to its unique role in gastrointestinal-associated functions, especially maintaining gut barrier integrity. Butyrate functions by promoting epithelial tight junctions, serving as fuel for colonocyte ATP production, and modulating the immune system. Interaction with the immune system occurs locally in the gastrointestinal tract and systemically in the brain. Investigation into research conducted on butyrate production pathways and specific bacterial players involved highlights a unique risk associated with use of gram-positive targeted antibiotics. We review and discuss evidence showing the relationship between the butyrate-producing gram-positive genus, *Roseburia*, and susceptibility to commonly prescribed, widely used gram-positive antibiotics. Considering gut microbiome implications when choosing antibiotic therapy may benefit health outcomes in patients.

## Introduction

Over 100 years ago, Nobel laureate Elie Metchnikoff proposed the idea that lactic acid bacteria are beneficial to human health.^1^ Since then, research into the human gut microbiome has grown exponentially. Nowadays, bacterial constituents of a microbial population can be identified by 16S rRNA-encoding gene sequencing.^2^ Additionally, “omics” methodologies such as transcriptomics, proteomics and metabolomics can provide information at successive levels of microbial physiology.^3^ Despite substantial advancements in our understanding of the bacterial portion of the normal microbiome, there are still a significant number of unknowns, including mechanistic relationships, unclassified gut bacterial species, and specific roles these bacteria may play in disease pathogenesis.

The European Metagenomics of the Human Intestinal Tract (MetaHIT)^4^ and the Human Microbiome Project (HMP)^5^ have provided a tremendous expansion of information and data in an effort to characterize the human microbiome. Characterization of bacteria that comprise a healthy human microbiome is an important initial step in understanding and determining the role of microbiome contribution to health and disease state. Despite significant efforts and research in this area, results support the idea that the gut microbiome is quite diverse compared to other body sites, and that there exists a considerable level of variation in the constituents of the gut microbiome in healthy individuals.^6^ While no true agreement has been reached, researchers and recent studies support the idea that roughly 70% of fecal bacterial species within an individual are stable for over 1 year, and few additional changes are observed out to 5 years.^7^ Calculations show that species are likely stable over decades, and potentially over an individual’s lifetime.^7^

Numerous studies have found an association between alterations in gut microbiome composition and a variety of diseases,^8-10^ although the mechanisms by which these associations occur is still largely unknown. It is often difficult to differentiate between cause and effect when studying the microbiome and disease. Evidence supports that diseased states alter gut microbiome composition and function through various mechanisms,^11^ but can the bacterial portion of the gut microbiome be a causative agent in human disease? It is important to properly elucidate relationships between gut microbiome alterations and human cell processes to determine true cause versus effect. Identification of bacterial gut microbiome constituents responsible for causing disease could allow for improvement in not only diagnostics but prevention and disease intervention.

Recently, bacterial production of short-chain fatty acids (SCFAs), specifically acetate, propionate, and butyrate, have gained a lot of attention in the research literature as they are shown to play a role in regulation of several physiologic systems in the human host including neuroinflammation within the nervous system,^12^ gluconeogenesis and appetite within the liver and pancreas,^13^ as well as blood pressure and coagulation within the cardiovascular system.^14^ By focusing on SCFA production and their interactions within the gastrointestinal tract and immune system, new hypotheses on functional relationships and interactions can be proposed to further elucidate their role in maintaining proper health. Previous primary research and large-scale reviews have broadly focused on gut microbiome produced SCFAs as a group, with little distinction between acetate, propionate, and butyrate as individual agents. In this review, we comprehensively delineate the roles of three individual SCFAs including butyrate, acetate and propionate, with emphasis on receptor affinity, involvement with signaling pathways, as well as net physiologic effects on the host. Once specific roles are delineated it is possible to look at external factors that may positively or negatively impact SCFA production and in turn, host health.

## Gut microbiome

Gut microbiota or the gut microbiome (GM) refers to the diverse microbial community that colonizes the human gastrointestinal (GI) tract. While termed gut microbiome, it does not only contain bacterial species but also fungi, viruses, and helminths.^15–17^ Bacteria comprise a significant portion of the gut microbiome and are well-characterized compared to the other “dark horses” that contribute to the overall microbiome. These other biomes; virome (viruses), mycobiome (fungi) and archaeome (archaea), and helminths are far less characterized and remain incompletely elucidated.^15^ Even less understood yet is the relationship and interactions between the various gut biomes. A study by García-Gamboa et al. found alteration in both the gut bacterial and fungal populations in healthy patients compared to those patients with obesity.^16^ While changes in composition have been observed in disease states, the relationship between those imbalances and the functional implications at the gut level are yet to be fully understood.^16^ The gut microbiome has been estimated to contain roughly 40 trillion bacterial cells, significantly outnumbering human cells in the body.^18^ The GM performs many functions vital to human physiology and survival, and many refer to it as “the forgotten organ”.^18^

The GM and its products are directly adjacent to the epithelial cells within the GI tract and alterations in its composition or metabolism may lead to changes in gut permeability and intestinal barrier function.^19^ This affects GI epithelial cells, immune cells, and the closely associated enteric nervous system.^20^ Research supports a bidirectional communication system between the gut microbiota and the brain, recognized as the “microbiota-gut-brain axis”.^21,22^ Additionally, gut bacteria may activate the immune system through a defective gut barrier, thus causing a systemic inflammatory response that can promote disease states, such as the increased blood–brain permeability seen in neuroinflammation.^23^

Gut microbes perform key functions for human health including energy extraction, biosynthesis of vitamins, protection against pathogen overgrowth, and education of the immune system.^18^ Microbial colonization of the gut occurs during birth, is highly dynamic through infancy, and resembles adult structure by about 3 years of age.^24^ Thereafter, the composition of the microbiome within an individual remains generally stable but is highly variable between individuals. Bacteria from the phylum Firmicutes and Bacteroidetes form a significant proportion (~90%) of the adult gut microbiota, while Actinobacteria composes the remaining.^25^

Sex, age, exercise, lifestyle, drugs, and diet can all affect the composition and functionality of the gut microbiome.^26^ Diet has been shown to promote the growth of specific microbiota patterns referred to as enterotypes, and long-term diet was shown to be strongly associated with enterotype partitioning.^27,28^ Enterotype 1, seen in industrialized countries, shows *Bacteroides* abundance and is typically associated with high fat, low fiber diets, with large quantities of refined foods, red meats, and dairy products. Enterotype 2 has *Prevotella* abundance and is typically associated with high fiber diets with less meat and dairy products. Some perspectives include enterotype 3, a makeup with *Ruminococcus* and co-occurring *Akkermansia* abundance, however, many classify this profile within enterotypes 1 for simplicity.^26,29,30^ Enterotype 2 is shown to produce higher SCFA compositions compared to *Bacteroides* dominant microbiota, due to more efficient fiber utilizing capacity.^31,32^ However, more research is needed to better understand the specific impact of enterotypes on health and disease states.

## Short-chain fatty acids

Much of the GM’s effects on host physiology and homeostasis are due to production of metabolites derived from bacterial fermentation.^33^ SCFAs, formic acid (C1), acetate (C2), propionate (C3), butyrate (C4), isobutyric acid (C4), isovaleric acid (C5), and valeric acid (C5) are produced through bacterial anaerobic fermentation of fibers or nondigestible carbohydrates such as resistant starch, simple sugars, and polysaccharides that cannot be digested or absorbed in the small intestine.^34,35^ Fermentation reactions can generate SCFAs as well as some gases, such as hydrogen gas, carbon dioxide and methane.^36^ SCFAs can exert both detrimental and beneficial effects on the GI tract and in distant sites throughout the body, such as the liver,^37^ pancreas,^38^ and brain.^16^ Acetate, propionate, and butyrate typically account for 95% of human production of SCFAs; this being roughly 60% acetate, and 15–25% both butyrate and propionate.^39,40^ Therefore, acetate, propionate, and butyrate will be the focus of this review, although we urge other researchers to continue examining the impact of the other SCFAs on host function.

**Figure 1.**
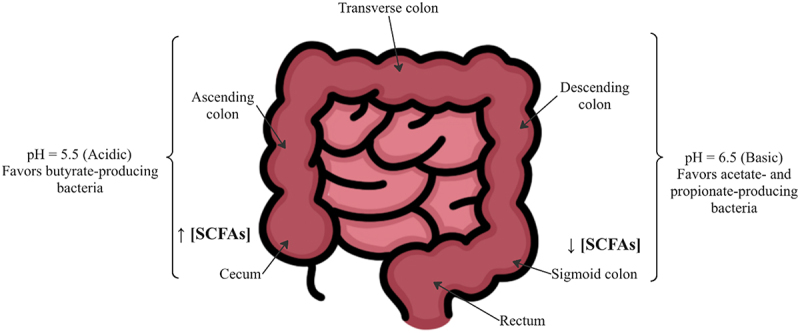
Gastrointestinal tract pH and concentration of SCFAs. The illustration shows a more acidic pH in the proximal colon and a less acidic pH in the distal colon. Additionally, there is a higher concentration of SCFAs in the proximal colon, where more bacterial fermentation occurs, and a lower concentration of SCFAs in the distal colon.

Fermentation primarily takes place on the proximal large intestine (cecum and ascending colon) and 95% of the SCFAs produced are rapidly absorbed by colonocytes.^34^ As luminal contents move from the proximal to distal colon, the concentration of available dietary fibers that can undergo fermentation for SCFA production decreases.^41^ Due to the imbalance of dietary fibers available for fermentation and subsequent production of SCFAs, the pH in various areas of the colon differs. In the cecum and proximal colon, where SCFA concentration is highest, the pH is more acidic, and in the distal colon, sigmoid and rectum, where SCFA concentration is lower, the pH is more basic.^42^ Human fecal microbiome studies show at a pH of 5.5 in the proximal colon that butyrate-producing bacteria, such as *F. prausnitzii* and *Roseburia* spp., comprise 20% of the total population.^43^ However, when luminal pH increases to 6.5 in the distal colon, butyrate-producing bacteria almost completely disappear, and acetate- and propionate-producing bacteria dominate.^44^ (Figure 1).

**Figure 2.**
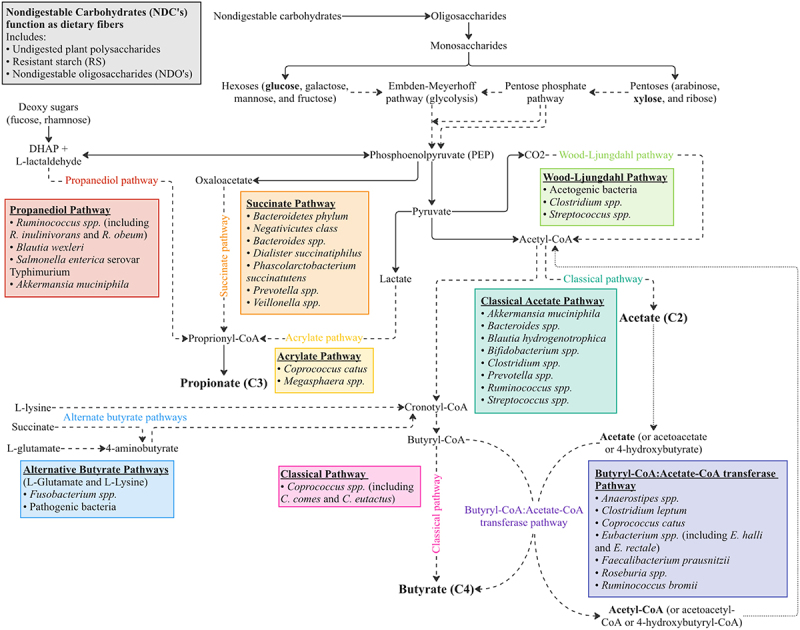
SCFA production pathways. Simplified schematic shows the various SCFA production pathways and the specific bacteria utilizing each pathway. Dashed lines represent the notion that more steps are involved but have been omitted for clarity. Colored text represents the named pathways with the corresponding colored box indicating the specific bacteria shown to utilize the respective pathway.

SCFA production involves multiple pathways, some of which are shared between species, and others unique to specific species. The pathways utilized by specific bacteria to make butyrate, propionate and acetate are described in detail in Table 1 and depicted in a simplified manner in Figure 2.^44–48^ The bacteria included are those that have been shown in previous studies to contain the enzymes necessary to produce the final product or intermediate metabolites in each pathway. It is likely that many other bacteria play a role in the production pathways; however, research in this area is lacking and should be investigated further.

**Table 1.** In-depth description of the steps, enzymes, and substrates involved in each pathway represented in Figure 2.

Pathway	Description of enzymes and steps involved	Sources	Bacteria
**Acetate (most acetate is produced by enteric bacteria)**			
Classical Pathway	Acetyl-CoA hydrolyzed to acetate by an Acetyl-CoA hydrolase	43–46	*Akkermansia muciniphila*^43 45^ *Bacteroides spp*.^43^ *Bifidobacterium spp*.^43 45^ *Blautia hydrogenotrophica*^43^ *Clostridium spp*.^43^ *Prevotella spp*.^43^ *Ruminococcus spp*.^43^ *Streptococcus spp*.^43^
Wood-Ljungdahl Pathway	Reduction of CO_2_ generates CO, which reacts with CoA and methyl group to produce acetyl-CoA which can be converted to acetate	43–46	*Acetogenic bacteria*^43^ *Clostridium spp*.^43^ *Streptococcus spp*.^43^
**Propionate (more conserved and substrate specific)**			
Succinate Pathway	Primitive electron transport chain (ETC) using PEP can be utilized to generate propionate. PEP is carboxylated to oxaloacetate, which is sequentially converted into malate and fumarate. Fumarate accepts electrons from NADH using fumarate reductase and NADH dehydrogenase, which form a simple ETC. NADH dehydrogenase transports protons across the cell membrane which are utilized for chemiosmotic ATP synthesis. Fumarate reductase also generates succinate. When the partial pressure of CO_2_ is low, succinate is transformed to methylmalonate, which leads to propionate and CO_2_. CO_2_ can be recycled for PEP carboxylation	43–46	*Bacteroidetes phylum*^43^ *Negativicutes class*^43^ *Bacteroides spp*.^46^ *Dialister succinatiphilus*^46^ *Phascolarctobacterium succinatutens*^46^ *Prevotella spp*.^46^ *Veillonella spp*.^46^
Acrylate Pathway	Reduces lactate to propionate by a lactoyl-CoA dehydratase. Pathway only present in a very limited number of gut bacteria	43–46	*Coprococcus catus*^43^ *Megasphaera spp*.^46^
Propanediol Pathway	Deoxy sugars, fucose and rhamnose, can be converted to 1,2-propanediol. This is sequentially converted to propionaldehyde and propionyl-CoA which is finally converted to propionate	43–46	*Akkermansia muciniphila* [major propionate producing species]^43,45^ *Blautia wexleri*^46^ *Roseburia inulinivorans*^43^ *Ruminococcus obeum*^46^ *Salmonella enterica* serovar *Typhimurium*^43^
**Butyrate (more conserve and substrate specific)**			
Classical Pathway	Phosphotransbutyrylase and butyrate kinase enzymes convert butyryl-CoA to butyrate-phosphate and then to butyrate	43–46	*Coprococcus spp*.^43^ *Coprococcus comes*^42^ *Coprococcus eutactus*^42^
Butyryl-CoA:Acetate-CoA Pathway	Butyryl-CoA:Acetate-CoA transferase enzyme converts butyryl-CoA into butyrate and acetyl-CoA from exogenously derived acetate. Preferred pathway in human gut microbiota	43–46	*Anaerostipes spp*.^45,46^ *Clostridium leptum*^45^ *Coprococcus catus*^46^ *Eubacterium rectale*^42,43,45^ *Eubacterium halli*^42,43,45^ *Faecalibacterium prausnitzii*^42,43,46^ *Roseburia spp*.^45^ *Ruminococcus bromii*^43^
Alternate Butyrogenic Pathways	Utilizes glutamate and lysine pathways. Results in release of harmful byproducts such as ammonia	43–46	*Pathogenic bacteria*^42^ *Fusobacterium*^42^

SCFAs exert effects throughout the body and the interplay is vast and too expansive for a single review. For the purposes of this review, the focus is on the effects within the GI epithelium, brain, and immune cells. Table 2 lists the receptor family name, the various receptors within that family, as well as the other names frequently used for the same receptor.

**Table 2.** SCFA receptors, ligands, and tissues expressing the receptors.

Receptor*	Ligand(s)	Tissue mRNA Expression (nTPM)^a^	Protein Expression^b^	Sources
GPR42 (FFAR3L, GPR41L, GPR42P)	Mainly activated by long-chain fatty acids (>12 carbons)	Adipose tissue (0.5) Colon (0.3)	Data not provided	45,46
GPR40 (FFA1R, FFAR1)	Mainly activated by medium-chain fatty acids (6–12 carbons)	Bone marrow (3.0) Pancreas (2.4) Spinal cord (2.3) Midbrain (1.1) Hippocampal formation (1.1) Basophil (1.0)	Data not provided	46
FFAR3 (FFA3R, GPR41)	Mainly activated by butyrate and propionate (activated to a lesser degree by acetate)	Adipose tissue (3.9) Appendix (2.4) Colon (1.2) Neutrophil (2.8) Eosinophil (2.5)	Duodenum^L^ Colon^L^ Small intestine^L^ Rectum^L^	44–47,49
FFAR2 (FFA2R, GPR43)	Mainly activated by acetate and propionate(activated to a lesser degree by butyrate)	Bone marrow (20.0) Appendix (17.3) Adipose tissue (4.7) Small intestine (4.5) Duodenum (3.2) Liver (1.5) Stomach (1.2) Neutrophil (249.8) Eosinophil (85.0) Non-classical monocyte (6.5) Intermediate monocyte (5.6) Basophil (3.1) Classical monocyte (2.1)	Bone marrow^M^ Duodenum^n/d^ Colon^n/d^ Small intestine^n/d^ Rectum^n/d^	44–47,49
GPR109a (HCAR2, HCA2, HM74A, NIACR1,Puma-g, PUMAG)	Mainly activated by butyrate	Bone marrow (21.9) Adipose tissue (21.4) Thymus (10.2) Tonsil (9.9) Appendix (9.6) Liver (2.8) Stomach (1.5) Small intestine (1.0) Neutrophil (177.3) Basophil (38.8) Non-classical monocyte (24.0) Intermediate monocyte (5.3) Eosinophil (2.6) Classical monocyte (2.5) Myeloid DC (1.7) Total PBMC (1.4)	Lymph node^M^ Bone marrow^M^ Duodenum^L^ Colon^L^ Small intestine^n/d^ Rectum^n/d^	44,45,47,50,51

SCFA receptor characteristics. This table includes a summary of SCFA receptors, their ligand affinity and relative tissue expression to be specific about the topic. Full table can be found in supplemental material; Supplemental Table 2. *Additional information regarding the various names of receptors can be found in supplementary table S1. ^a^Only tissues with transcription per million (nTPM) ≥ 1.0, or the top 3 tissues with the highest expression are shown. ^b^Protein expression classified as high with superscript “H”, medium with superscript “M”, low with superscript “L” or not detectable with “n/d”. Monocyte subsets^52^ are discussed in further detail in the section “SCFAs and the immune system.”

Prior to discussing the physiologic role of SCFAs, it is important to understand the variable expression of the SCFA receptors. Table 2 shows the receptor, preferred ligand, mRNA expression, and protein expression.

## GI epithelium and colonocytes

**Figure 3.**
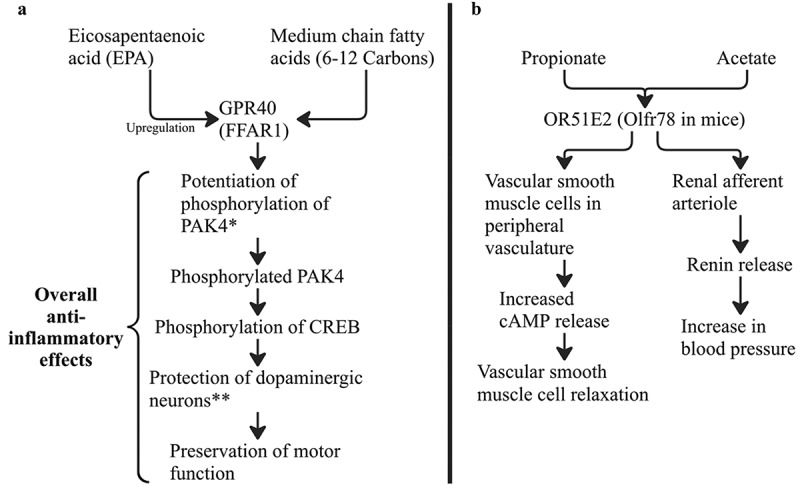
(a) GPR40 receptor and activity. Anti-inflammatory effects seen in the central nervous system through activation of GPR40 receptor.^57^ CREB: cAMP-response element-binding protein; PAK4: p21-activated kinase 4. *in mouse islet cells. **in rat model of PD. (b) OR51E2 receptor and activity. OR51E2 is activated by acetate and propionate and the associated physiologic effects of receptor activation include vascular smooth muscle relaxation and increased blood pressure.

SCFAs act as signaling molecules within GI epithelium, as well as other tissues throughout the body via interactions with several known receptors. GPR40 has been recognized as a medium-chain fatty acid receptor and GPR42 as long-chain fatty acid receptor.^53^ GPR40 is located in various tissues and organs such as the brain, pancreas, spinal cord, and heart.^54,55^ Multiple previous studies have shown that activation of GPR40 receptor by medium-chain fatty acids has beneficial anti-inflammatory effects and provides neuroprotection after central nervous system injury^56^ (Figure 3a). The function and expression of GPR42 is less well described, and current research suggests that it may be the result of a gene duplication event of GPR41.^58^

The receptor Olfr78 in mice, OR51E2^59^ being the human equivalent, mainly binds acetate and propionate and is expressed on vascular smooth muscle cells of peripheral vasculature and the renal afferent arteriole.^60^ In vascular smooth muscle cells, binding of acetate or propionate to the receptor leads to an increase in cyclic adenosine monophosphate (cAMP), a second messenger with a wide variety of possible effects.^61^ In renal afferent arteriole cells, binding of acetate or propionate results in an increase in renin release which can contribute to an increase in blood pressure^61^ (Figure 3b).

**Figure 4.**
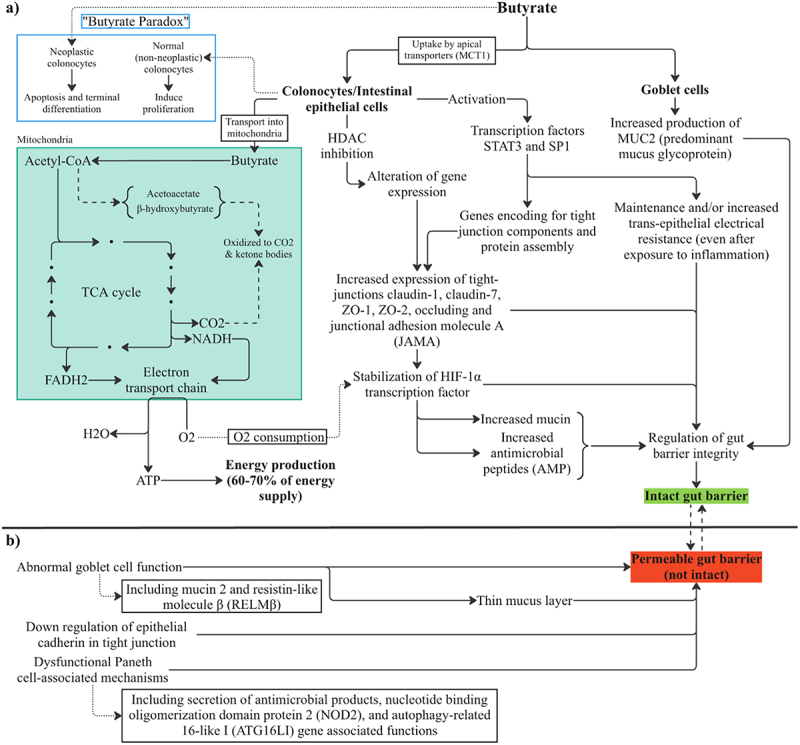
(a) Butyrate action in gut epithelial cells; colonocytes, goblet cells, and neoplastic versus non-neoplastic colonocytes. Butyrate supports host homeostasis by promoting proliferation of non-neoplastic colonocytes, while inducing apoptosis in neoplastic colonocytes and regulating multiple signaling pathways. (b) factors impacting the balance between gut barrier integrity and breakdown. Butyrate promotes gut barrier integrity by increasing expression of tight junction proteins, mucin 2 production, and stabilization of HIF-1. Additional source for factors contributing to breakdown mechanisms of the gut barrier have been reported.^64^ HDAC: histone deacetylase.

**Figure 5.**
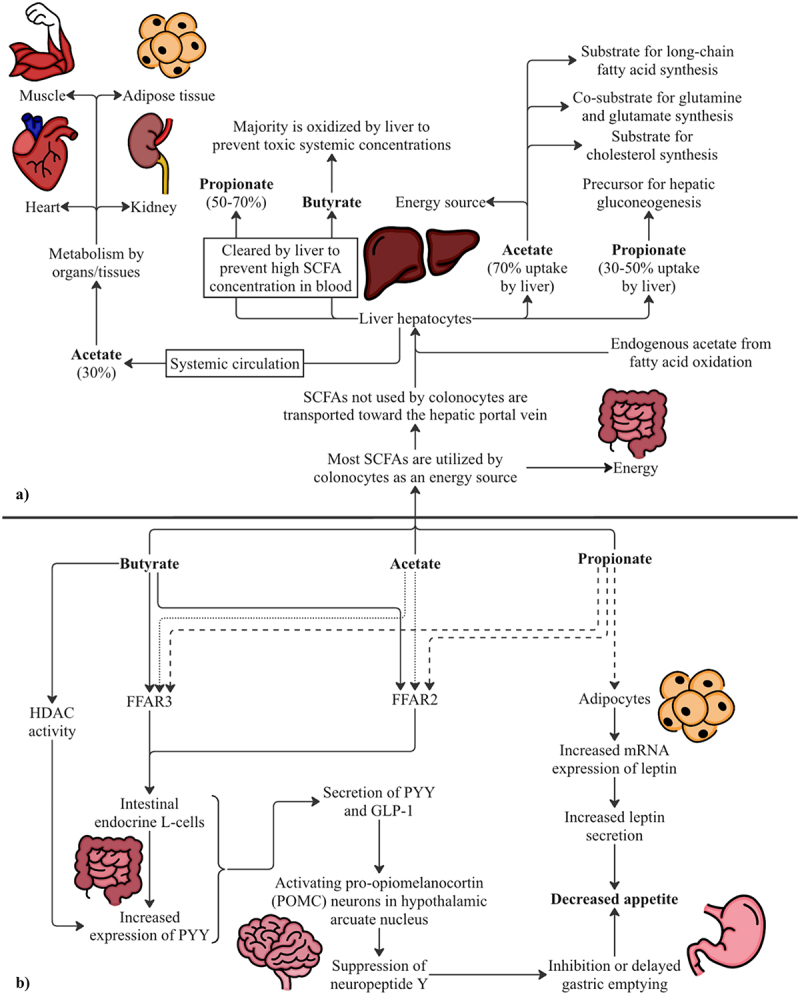
(a) SCFA utilization beyond the gut epithelium. SCFAs not utilized by colonocytes are taken up by hepatic portal system, transported to the liver and have various fates. Butyrate is mainly oxidized by the liver and minimal amounts from the GI tract enter systemic circulation. (b) the role of SCFAs in energy balance. To clarify the overlap and variation between the SCFAs and their binding targets, solid lines are used for butyrate, dashed line for propionate and dotted lines for acetate. After receptor binding, solid lines are used for all subsequent steps. SCFAs act through FFAR2 and FFAR3 receptors to produce PYY and GLP-1 which act centrally to decrease appetite.

Most SCFAs are utilized by colonocytes as an energy source, with butyrate being the primary source.^62^ Specifically, colonocytes are estimated to derive 60–70% of their energy supply from SCFA oxidation.^63^ Research has shown that colonocytes prefer butyrate to acetate or propionate for oxidation to ketones and CO_2_ in generation of energy^63^ (Figure 4a). This is based on colonocytes displaying a relatively high affinity for butyrate; however, studies on human and rat colonocytes show maximum flux of butyrate being 0.4 µmol/min/g cell weight and 0.6 µmol/min/g cell weight for acetate.^65,66^ Thus, under conditions where colonic concentration of acetate is greater than that of butyrate, acetate may serve just as important of a role as butyrate for colonocyte energy supply. SCFAs not utilized by colonocytes enter the hepatic portal circulation and are transported to the liver. About 70% of acetate is taken up by liver and used for various functions (Figure 5a), whereas the remainder enters systemic circulation and is metabolized by other tissues, such as heart, adipose tissue, kidney, and muscle.^67,68^ To prevent elevated levels of SCFAs in the blood, most propionate and butyrate are cleared from portal circulation by the liver.^68^ Propionate can act as a precursor to gluconeogenesis in hepatocytes with research suggesting that 30–50% is taken up by the liver.^68^ Peripheral tissues take up the remainder of propionate available in the systemic circulation.^68^ The small amount of butyrate that reaches it is largely taken up by the liver and oxidized by hepatocytes to prevent toxic systemic concentrations.^68^ Overall, the SCFAs produced by the gut microbiome are utilized within the colonocytes and very small amounts of SCFAs reach the liver with even fewer reaching systemic circulation.

Interestingly, research has shown that butyrate can induce proliferation in normal colonocytes but also terminal differentiation and apoptosis in neoplastic cells. This dual role has been termed the “butyrate paradox”.^69^ In respect to gut barrier integrity, research has shown that butyrate taken up by colonocytes can strengthen barrier integrity by increasing gene expression of tight junction proteins such as claudin-1, claudin-7, ZO-1, ZO-2, occluding, and junctional adhesion molecule A (JAMA).^70^ One group of researchers showed that butyrate promotes barrier integrity through activation of genes encoding tight junction components and protein assembly through transcription factors STAT3 and SP1.^47^ The activation of these various genes and upregulation of tight junction proteins results in the maintenance and/or increased transepithelial electrical resistance (TEER) in humans, even after exposure to inflammatory conditions.^71^ Furthermore, since butyrate is the preferred source of oxidative fuel, its uptake and metabolism by colonocytes contributes to oxygen consumption and maintenance of an anerobic environment which contributes to the stabilization of hypoxia inducible factor-1α (HIF-1α). In the gut epithelium, stabilization and transcriptional activity of HIF-1α is protective to gut barrier integrity.^72^ HIF-1α transcription factor has been shown to increase expression of mucin (MUC), antimicrobial peptides (AMP), and tight junction proteins.^73^ Butyrate is also able to modulate mucus layer thickness via increased secretion of MUC2, a predominant mucin glycoprotein, by Goblet cells^74^ (Figure 4b).

Recent research shows that SCFAs not utilized by colonocytes may impact the body’s mechanisms of energy intake and energy use through FFAR3 and FFAR2 receptor activation in intestinal enteroendocrine L-cells.^75^ These cells are responsible for the production and secretion of gut-derived satiety hormones peptide YY (PYY) and glucagon-like peptide-1 (GLP-1).^76^ Both GLP-1 and PYY influence appetite and satiety by activating pro-opiomelanocortin (POMC) neurons in the hypothalamic arcuate nucleus which results in the suppression of neuropeptide Y (NPY).^77^ Suppression of NPY in the brain results in delayed or inhibited gastric emptying.^78^ Additionally, butyrate’s inhibitory action on HDAC leads to an increased expression of PYY in human L-cells.^79^ Beyond the GI tract, propionate has been shown to interact with adipose tissue causing increased mRNA expression and secretion of leptin.^80^ Together, these mechanisms may influence satiety and cause decreased appetite (Figure 5b).

## SCFAs and the immune system

Information presented in Table 2 brings up interesting potential connections when analyzing receptor expression on immune system cells, specifically different subsets of monocytes, and the functional roles of these cells. Monocytes can be divided into subsets based on CD14 and CD16 expression, with different subsets exhibiting distinct functional properties.^52^ Tissue mRNA expression shows that FFAR2 receptors are more predominantly expressed on non-classical monocytes and to a lesser degree on intermediate monocytes, than on classical monocytes. Similarly, GPR109a expression has significantly higher expression on non-classical monocytes, than the other subsets, but can still be found expressed on intermediate and classical monocytes to a lesser degree.

It is widely accepted that classical monocytes can differentiate into monocyte-derived macrophages and DCs, likely playing an integral role in shaping inflammation and resolution of inflammation in tissues.^81,82^ This subset of monocyte has been shown to contribute to chronic disease.^80^ Classical monocytes were found to be primed for phagocytosis, innate sensing/immune responses and migration.^49^ This subset of cells is recruited during bacterial infections to sites of inflammation and function to recognize and phagocytose pathogens, secrete proinflammatory cytokines, as well as recruit other immune cells for regulation of the inflammatory response.^82-85^ Classical monocytes migrate to CCL2 and CCL3 gradients and are more efficient than intermediate monocytes at producing reactive oxygen species (ROS) and containing fungi.^86-88^ In humans, classical monocytes express higher levels of chemokine receptors such as CCR1, CCR2, CCR5, CXCR1, and CXCR2.^87,89^ This finding highlights their potential to migrate to areas of injured or inflamed tissues. Additionally, this subset is also characterized by their ability to secrete pro-inflammatory molecules such as IL-6, IL-8, CCL2, CCL3, and CCL5.^89,90^

Intermediate monocytes have comparable ROS production and phagocytosis potential to classical monocytes.^52^ However, intermediate monocytes showed lower adhesion to surfaces and higher class II molecule expression and IL-12 production than classical monocytes.^52^ Additionally, this subset of monocytes expresses the highest levels of antigen presentation-related molecules and are shown to secrete TNF-α, IL-1β, IL-6, and CCL3 upon TLR stimulation.^89–92^ Intermediate monocytes are the only subset expressing CCR5 and thus well-suited for antigen presentation, cytokine secretion, apoptosis regulation, and differentiation.^50^ This increased expression of CCR5 also likely accounts for their increased susceptibility to HIV-1 infections.^87–94^ Higher levels of intermediate monocytes are found in the blood of patients with systemic infections, suggesting they may play a role in the rapid defense against pathogens.^95,96^ However, another report found that this subset of monocytes are the main producers of IL-10 upon TLR stimulation.^97^ These conflicting results contribute to the unclear nature of their exact role in immunity and inflammation.

Non-classical monocytes also have antigen processing capabilities but are distinguished from other subsets by their association with the wound healing process.^98^ They are involved in complement and Fc-mediated phagocytosis and adhesion.^52^ Non-classical monocytes have been widely viewed as anti-inflammatory, as they maintain vascular homeostasis.^82^ These cells have an antagonizing function to the classical monocytes and promote neutrophil adhesion at the endothelial interface via the secretion of TNF-α and do not reach the same production level of pro-inflammatory cytokines as classical monocytes.^99,100^ Non-classical monocytes are referred to as “patrolling monocytes” because the cells show a distinct motility and crawling pattern along vasculature and function to survey the luminal side of vascular endothelium in an LFA-1- and integrin-α_4_-dependent manner.^101^ Additionally, this subset of monocytes recognizes and clears dying endothelial cells in a TLR7-dependent manner to maintain vascular homeostasis.^102,103^

Human monocyte differentiation and function in the gut is influenced by dietary components, host metabolites and bacterial-derived products.^104–106^ Bacterial-derived butyrate imprints a host protection program via epigenetic remodeling during differentiation of monocytes to macrophages in the lamina propria.^107^ Specifically, without tissue-damaging inflammation, butyrate induces macrophages to upregulate antimicrobial proteins in the gut such as calprotectin.^107^ Thus, in states of depleted butyrate and excess acetate, it is possible that activation of monocytes and binding via FFAR2 is more prominent than via GPR109a. A study by Ang et al. showed treatment of monocyte subsets with the SCFA acetate resulted in human monocytes with elevated p38 phosphorylation^108^ which has been shown to promote differentiation of monocytes into M1 inflammatory macrophages.^109^ Interestingly though, acetate signaling through FFAR2 also showed attenuation of C5, CCL1, CCL2, GM-CSF, IL-1α, IL-1β, and ICAM-1 inflammatory cytokine expression.^108^ SCFA acetate also repressed Akt and ERK2 signaling.^106^ Activation of the PI3K/Akt pathway is critical in restricting proinflammatory and promoting anti-inflammatory responses in TLR-stimulated macrophages.^110^ MEK/ERK signaling is involved in the activation of oxidative and nitrosative bursts, endosomal trafficking, and proinflammatory macrophage polarization.^111^ Taken together, suppression of these pathways could help potentiate a dysregulated immune response and potentially a proinflammatory-biased environment.

**Figure 6.**
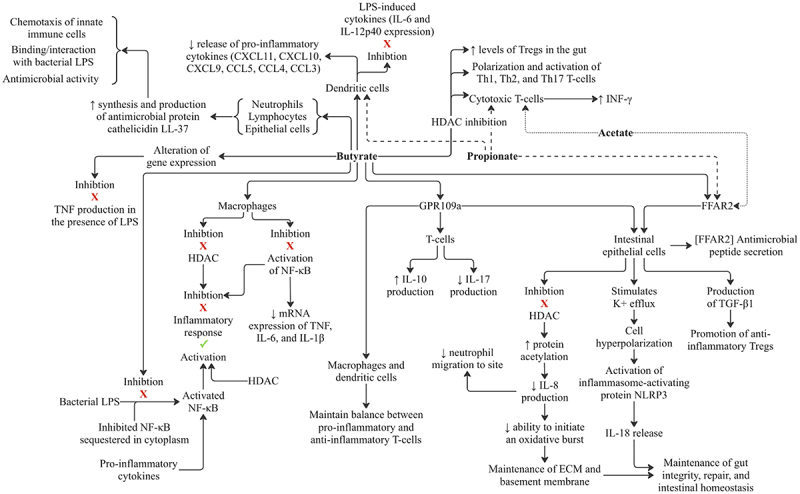
SCFAs and their role in the innate and adaptive immune system. Butyrate has vast and varying influences on branches of the immune system, including innate and adaptive immunity. While acetate and propionate can interact with aspects of the immune system, they possess far fewer routes of immunomodulation compared to butyrate. LPS: Lipopolysaccharide; HDAC: histone deacetylase; Tregs: T-regulatory cells.

SCFAs, mainly butyrate, have been shown to interact with the immune system in numerous ways (Figure 6). Butyrate has been shown to inhibit HDAC^112^ and the activation of NF-κB (nuclear factor kappa-B) in macrophages.^113^ Both HDAC and NF-κB are known to be involved in the inflammatory response.^114^ NF-κB consists of a heterodimer and is sequestered by inhibitory proteins in the cytoplasm in non-diseased states.^115^ Research has shown that NF-κB can be activated by proinflammatory cytokine, bacterial LPS, or variety of other stimuli.^115^ Recent research using biopsied cell cultures obtained from Crohn’s disease (CD) patients showed that butyrate can inhibit LPS-induced NF-κB activation and gene transcription.^116^ These researchers also found that butyrate had a dose-dependent effect on TNF levels. At 10 mM of butyrate, TNF levels, IL-6 levels and IL-1β levels returned to healthy control-level values. At doses as low as 2 mM of butyrate, it strongly inhibited LPS-induced TNF production by peripheral blood mononuclear cells in both CD patients and control patients. Butyrate was also shown to act in a dose-dependent manner on mRNA expression of cytokines in lamina propria mononuclear cells and colonic mucosal cells. LPS induced TNF-β, IL-1β and IL-6, which all increased in CD patient biopsies. Butyrate treatment resulted in reduction or inhibition of LPS-stimulated expression of cytokine mRNA. Butyrate has also been shown to manipulate gene expression in neutrophils, a mechanism that is thought to inhibit TNF production in the presence of LPS.^117^ SCFAs, mainly acetate and propionate, can also promote antimicrobial peptide secretion through interaction with intestinal epithelial cells and FFAR2 receptor.^118^

Additionally, SCFA binding to FFAR2 and GPR109a on intestinal epithelial cells stimulates K^+^ efflux and hyperpolarization, causing activation of inflammasome-activating protein NLRP3 and induction of IL-18 release. Research shows that IL-18 helps with maintenance of integrity, repair, and intestinal homeostasis.^119^ Additionally, butyrate-induced inhibition of HDAC leads to increased protein acetylation and a decrease of IL-8 production in intestinal epithelial cells.^120^ IL-8 (CXCL8) is also known as neutrophil chemotactic factor and functions to induce chemotaxis in targets cells, primarily neutrophils but also other granulocytes, causing immune cell migration to the site.^121^ Another key function of the cell signaling stimulated by IL-8 is the initiation of the oxidative burst. This process allows the buildup of proteolytic enzymes and reactive oxygen species which are necessary to break down the ECM (extracellular membrane) and basement membrane.^122^

In dendritic cells, butyrate and propionate have been shown to decrease the release of pro-inflammatory cytokine CXCL11, CXCL10, CXCL9, CCL5, CCL4, and CCL3, as well as inhibiting expression of LPS-induced cytokine IL-6 and IL-12p40.^123^ Butyrate is known to contribute to host defense by inducing the anti-microbial protein cathelicidin LL-37 in the gut.^124^ Cathelicidin LL-37 is an antimicrobial peptide synthesized and secreted by epithelial cells, neutrophils, and lymphocytes.^124^ This molecule serves a triple function for chemotaxis of innate immune cells, anti-microbial activity, and interacting with negatively charged molecules, such as the LPS component of gram-negative bacteria and teichoic acid of gram-positive bacteria.^125^

Macrophages and dendritic cells express GPR109a receptors, and activation of this receptor by butyrate is demonstrated to be crucial in maintaining the balance between pro-inflammatory and anti-inflammatory T-cells.^126^ Studies performed in GPR109a knock-out mice show reduced IL-10 and elevated IL-17 production by T-cells, leading to increasing susceptibility to colitis.^126^

Butyrate has also been proven to increase the levels of T-regulatory cells in the gut^127^ and facilitate extrathymic peripheral polarization toward regulatory T-cells in vitro and in vivo.^128^ Butyrate-producing Clostridia spp. have been shown to induce colonic regulatory T-cells in mice.^129^ The proposed causal mechanism is currently attributed to butyrate’s inhibitory effects on HDAC, where inhibition of HDAC is proposed to cause increases in regulatory T-cells.^128^ Interestingly, it has been observed that acetate does not provide the same inhibitory action of HDAC seen with butyrate.^130^ Butyrate can also induce production of TGFβ1 by intestinal epithelial cells, promoting anti-inflammatory regulatory T-cells (T-regs).^131^

Beyond regulatory T-cells, inhibition of HDAC by butyrate can also affect polarization and activation of Th1, Th2 and Th17 T-cells.^132^ Additionally, in cytotoxic T-cells, butyrate and propionate inhibition of HDAC has been shown to modulate gene expression by promoting INF-**γ** expression.^133^ Acetate is also suggested to enhance IFN-**γ** production, but by acting as a metabolic substrate for cytotoxic T-cells rather than directly influencing gene expression.^133^

Research substantially supports that the SCFA butyrate, specifically, has anti-inflammatory properties thought to be owing to an epigenetic mechanism^134^ or to the effects of SCFA receptors. It has also been shown that these receptor effects are anti-inflammatory, anti-microbial, and decrease intestinal barrier leakiness^126^ by maintaining tight junctions and preventing dysbiosis-associated gut permeability.^135^ Butyrate also alters the activity of enteric neurons by reversible hyperpolarization and reducing colonic contractility.^136,137^ Sodium-butyrate, a histone deacetylase inhibitor (HDACi), protects dopaminergic neurons^136,138^ and prevents motor impairment in a toxin-induced drosophila model of Parkinson’s Disease.^139^ In critically ill patients, lower concentrations of butyrate are associated with GI dysmotility.^140^ Colonizing germ-free mice with butyrate-producing bacteria *Clostridium tyrobutyricum* or oral administration of sodium butyrate upregulated tight junction protein function.^141^ Additionally, intragastric treatment of *Clostridium butyricum* shows improvement of neurological dysfunction, brain edema, neurodegeneration, and blood–brain barrier impairment in post-traumatic brain injury mouse models.^142^

This collection of research supports the notion that butyrate plays a significant role in many different systems within the host. Unlike the other SCFAs that are routinely associated with, acetate and propionate, alterations in butyrate levels seem to have a more substantial impact on host physiology, pathophysiology, and disease processes. Thus, it is possible that alterations in gut microbiome composition and function, specifically relating to butyrate-producing bacteria, may serve as pathologic drivers in various inflammatory and colonic-associated disease, or as a route to explore as preventative treatment in a variety of conditions.

## Future direction and clinical application – risk of antibiotics on SCFA production

The gut microbiome does not exist within a vacuum and is susceptible to numerous internal and external factors that can alter its composition and function. The most common external perturbations to the gut microbiome are diet, environment, and medications, specifically antibiotics.^143,144^ Individual microbiome response to antibiotics is highly variable and dependent upon the state of the microbiome at the time of perturbation and the strength of the perturbation.^145^ Antibiotics have revolutionized the treatment of infectious diseases and are widely prescribed with recent studies reporting continued increased consumption of antibiotics in certain countries in the past few years.^146^ However, researchers have recently shown the detrimental effect of broad-spectrum antibiotics, such as cephalosporins, on the gut microbiome.^147^

Understanding the intricacies of antibiotic bacterial efficacy can be complex and is an ever-changing landscape due to bacterial resistance mechanisms. Antibiotics are classified based upon mechanism of action, for example, penicillins and cephalosporins are both under the category of cell wall inhibitors.^148^ Additionally, penicillins and cephalosporins are referred to as beta-lactam antibiotics due to the structure of the medications containing a beta-lactam ring as a part of their chemical structure.^148^ Within the category of penicillins, there are four subclassifications which include, natural penicillins, aminopenicillins, penicillinase-resistant-penicillins, and antipseudomonal penicillins.^148^ While each of these subclassifications varies slightly in use and indications, the class of penicillin antibiotics are largely used for gram-positive infections.^148^ Cephalosporins are another larger group of cell wall inhibitor antibiotics and are broken down further into five generations.^148^ Each generation, 1^st^ through 5^th^, has a slightly varying spectrum of coverage and indications for use but are still broadly used for gram-positive bacterial infections.^149^ Amoxicillin is classified as an aminopenicillin antibiotic that is widely used in the primary care setting^150^ and effective against a wide range of gram-positive bacteria. Compared to natural penicillin, amoxicillin has some additional coverage against some gram-negative species.^150^ The oral formulation of Amoxicillin was reported to be the most prescribed antibiotic in an out-patient setting in 2022.^151^ Ceftriaxone is classified as a 3^rd^ generation cephalosporin.^149^ Ceftriaxone is a broad-spectrum antibiotic that is effective against a wide range of gram-positive and gram-negative infections.^149^ Ceftriaxone, administered via injection, was reported to be the most prescribed antibiotic in the inpatient setting,^152^ while cephalexin administered in an oral formulation was the most prescribed cephalosporin in out-patient setting.^151^ A breakdown of the antibiotic classifications described above can be found in supplemental Table 3.

**Table 3.** Monocyte characteristics as they relate to SCFA receptor expression.

Monocyte subset^52^	Surface markers^52^	FFAR2 expression	GPR109a expression
Classical monocytes	CD14^+^ CD16^–^	2.1 nTMP	2.5 nTMP
Intermediate monocytes	CD14^+^ CD16^+^	5.6 nTMP	5.3 nTMP
Non-classical monocytes	CD14^–^ CD16^+^	6.5 nTMP	24.0 mnTMP

Numerous studies have evaluated antibiotic use and its impacts on the microbiome, both short term and long term.^153–155^ Infants appear to be the most susceptible due to lack of a well-established gut microbiome at birth and microbiome maturation in the first years of life.^156^ The first years of life are incredibly important in developing a healthy microbiome profile.^157^ Early antibiotic administration has been associated with many health conditions, including increased BMI,^158^ wheezing,^159^ infantile colic.^160^ Even before birth an infant’s gut microbiome and future health can be affected by exposure to antibiotics in pregnancy, which has been associated with increased risk of Very Early Onset Inflammatory Bowel Disease (VEO-IBD).^161^ However, not all antibiotics are shown to impact the microbiome equally. In the example of broad-spectrum antibiotic use in neonates, which are indicated in the suspicion of early onset neonatal sepsis, researchers demonstrated amoxicillin + cefotaxime co-administration for 48 hours conveyed the largest effects on microbiome composition and antimicrobial resistance gene profile in the treatment group^162^ when compared to a control group of healthy, non-antibiotic treated infants. Researchers also assessed other antibiotic combinations such as penicillin + gentamicin and co-amoxicillin/clavulanate + gentamicin each with 48 hours of administration. Penicillin + gentamicin exhibited the least effects on microbial community composition and antimicrobial resistance gene profiles.^162^ Additionally, they found the genera *Bifidobacterium* to be reduced in the amoxicillin + cefotaxime group compared to the penicillin + gentamicin group.^162^ The study concluded that there are significant long-term side effects of broad-spectrum antibiotic use in treatment of infants with suspected early onset neonatal sepsis, despite most neonates’ antibiotics being stopped after 48 hours. They also found that the impact to microbial diversity, community composition, and antimicrobial resistance gene profiles were still measurable at 12 months of life.^162^

Mouse studies also consistently show significant shifts in microbiome profiles with a variety of antibiotic use, all sharing similar depletion of SCFAs butyrate, acetate, and propionate.^163–166^ Mouse studies show administration of ceftriaxone significantly reduces concentrations of SCFAs following withdrawal.^167^ Given the importance of butyrate to the intestinal barrier, as discussed above, it is no surprise that mouse models show antibiotic-induced dysbiosis with reduced levels of ZO-1, occludin, and claudin-1. Reduction of these proteins results in tight junction damage and increased intestinal permeability.^168^ Additionally, increased intestinal permeability due to alterations in tight junction protein expression may contribute to increased food sensitivity seen following antibiotic use.^168^ Research also supports the hypothesis that cognitive impairment secondary to antibiotic use is likely related to gut dysbiosis rather than systemic antibiotic response as demonstrated by antibiotic-induced distinct alterations in tight junction proteins and circulating metabolite levels similar to those of germ-free mouse models.^163^

## Potential mechanisms of microbiome shifts impacting butyrate production pathways

Given that medications are one of the most common sources of external perturbations, it can be established that oral administration of antibiotics has the potential to affect the gut microbiome composition, regardless of class or spectrum.^145^ Antibiotic presence in the gut, regardless of if the GI tract is the source of the infection or just the method of absorption of the antibiotic, can exert toxicity to the bacteria within its spectrum.

As shown in Figure 2 and Table 2, the Butyryl-CoA: acetate-CoA transferase pathway is the preferred butyrate production pathway in human gut microbiota. Importantly, this pathway utilizes mostly gram-positive bacteria. This is important because gram-positive bacteria are historically most susceptible to commonly used antibiotics focused on disrupting cell wall synthesis, most notably amoxicillin and the 1^st^ generation cephalosporin cephalexin. For example, *Roseburia*, one of the most abundant contributors to Butyral-CoA: acetate-CoA transferase pathways, has been shown to be very susceptible to oral amoxicillin/clavulanic acid treatment.^169,170^ Other major butyrate-producing bacteria have been shown to be reduced with amoxicillin including *Clostridium* and *Ruminococcus spp*.^171,172^ Thus, certain antibiotic use may have a unique preference for disrupting gut barrier integrity by promoting the reduction of important butyrate specific producing bacteria.

Since treatment with certain antibiotics can ultimately lead to decreased butyrate-producing species in the proximal colon, the niche can be filled by various other organisms depending on disease state, diet, and several other external factors. This includes the potential of recolonization by primarily acetate and propionate producing species or even pathologic bacteria. Recolonization by species such as *Lactobacillus* or *Bifidobacterium* may be possible by patient co-ingestion of antibiotics and probiotics. This can be expected since the seven core genera of microbial organisms most often used in probiotic products are *Lactobacillus*, *Bifidobacterium*, *Saccharomyces*, *Streptococcus*, *Enterococcus*, *Escherichia*, and *Bacillus*.^172–174^ It is also possible that recolonization by acetate- and propionate-producing species in the GI tract may have physiologic mechanisms that allow certain genera or species to recolonize these vacancies in a faster manner, possess mechanisms associated with accelerated doubling time, or may not be as susceptible to certain antibiotics. Regardless of method, if the vacated niche is recolonized by non-butyrate-producing species or species that prefer other SCFA production, the ability of butyrate-producing species to repopulate will be diminished, leading to the consequences observed with decreased butyrate in the GI tract.

Another potential mechanism for decreased butyrate-producing bacteria could be initiated by some form of gut microbiome dysbiosis of butyrate-producing bacteria secondary to external factors, such as a lack of prebiotics or diets high in simple sugars. Prebiotics are a source of food for the gut’s healthy bacteria, such as carbohydrates that cannot be digested by humans but instead are digested by gut microbes in the lower GI tract and act as food source for healthy commensal bacteria. Prebiotics are in foods such as whole grains, bananas, greens, onions, garlic, soybeans, and artichokes. Not consuming enough prebiotics can lead to dysbiosis, which has been linked to various GI problems such as IBS and related symptoms. Probiotics, on the other hand, are foods or supplements that contain live organisms^173,175^ and may also be implicated in external factors inducing dysbiosis. If patients were to supplement with commercially available probiotics, this could result in population/colonization of the proximal colon with primarily acetate and propionate producing bacteria. This would result in a change in the pH of the proximal colon and further inability for repopulation of butyrate-producing bacteria (due to pH, space occupied by other bacteria, etc).

Thus, it may be beneficial for patients to supplement antibiotic treatment with prebiotics and butyrate-producing specific bacterial probiotics. Thus, targeted probiotic therapy with butyrate-producing bacteria, such as *Roseburia*, may lead to a more efficacious oral microbiome reseeding protocol following common antibiotic treatments that deplete favorable butyrate-producing pathways. This may improve gut level integrity and reduce immune system activation in cases of certain antibiotic usage or other dysbiosis. Overall, *Roseburia* or other butyrate-producing bacteria focused probiotic protocols should be studied as a possible mechanism to help improve gut epithelial integrity.

Additionally, due to the positive gut protective effects of butyrate, isolated butyrate supplementation may be beneficial during the use of antibiotics until butyrate-producing bacteria are properly recolonized. Butyrate supplementation has already been utilized to improve intestinal mucosa integrity and as a tool for reducing inflammation in systemic diseases.^176-177^

Similar mechanisms can be seen for all other SCFA and their appropriate levels as well, but Butyrate has shown significant importance in its anti-inflammatory and immunoregulatory mechanisms. Also, due to *Roseburia* specific susceptibility, we argue butyrate production should be focused on further in research given the significant side effect of recurrent or excessive antibiotic use.

## Limitations

Microbiome research is historically complex, and consensus is continually changing. It is unknown if probiotics truly alter microbiome composition over extended use timelines, however, it has been proven that even if the composition of the gut microbiome doesn’t change, the administration of probiotics does impact the GI metabolome, conferring a positive overall effect.^178^ Specific prebiotic supplements are even less researched, so it is largely unknown what the long-term effects on the gut microbiome are, however, diet is a major external factor that has the potential to alter GM composition, so it is plausible that prebiotics may alter GM composition. Lastly, although we discuss oral probiotics in this review, oral probiotics are significantly less effective than fecal microbiome transplants in reversing antibiotic associated dysbiosis,^179^ thus fecal transplants of butyrate-producing bacteria may be a better strategy. However, fecal transplants are much more expensive and usually reserved for serious cases of *Clostridium difficile*, therefore, data would be needed to show efficacy in such cases.

## Conclusion

Many studies discuss SCFAs as a broad category, however, we propose it is important to start delineating butyrate, propionate, and acetate due to their unique receptor interactions, physiologic functions, and associated gut microbes. Although all SCFAs have some role in colonocyte energy supply, each SCFA can activate unique pathways in specific tissue types and promote different metabolic pathways. For example, butyrate possesses the ability to improve gut health and epithelial integrity by promoting tight junction protein expression and goblet cell-dependent mucin production. Additionally, it has well documented anti-inflammatory effects owing to extensive innate and adaptive immune cell interactions and regulation. As gut microbiomes and gut dysbiosis have gained increased attention in several diseased states, it is vital for clinicians and researchers to consider the potential negative iatrogenic effects of treatment plans. As discussed, antibiotics serve as an important mechanism for eliminating and treating bacterial infection, however, treatment with certain antibiotics may also negatively impact vital butyrate-producing bacteria. Antibiotic therapy can lead to increased gut permeability, gut-associated inflammation, as well as systemic inflammation. Thus, future research and treatment protocols should emphasize the importance of prophylactic or reseeding protocols for butyrate-producing bacteria for patients prescribed antibiotics, in order to reduce negative outcomes.

## Supplementary Material

Supplemental Material

## Data Availability

Data sharing is not applicable to this article as no new data was created or analyzed in this study.
