# Supplementary material for: The interplay between gut microbiota, short-chain fatty acids, and implications for host health and disease
Source: Gut Microbes. 2024 Sep 16;16(1):2393270. doi: 10.1080/19490976.2024.2393270 (PMC11407412; doi:10.1080/19490976.2024.2393270)
Supplement: Supplemental Material [file KGMI_A_2393270_SM1727.zip › Supplemental_Information_Butyrate_paper.docx]

Supplemental Information

Supplemental Table 1: Summary of SCFA receptors and synonymous names.

| Free Fatty Acid Receptor (FFAR) | G-Protein Receptor (GPR) | Hydroxycarboxylic Acid Receptor (HCAR) | Olfactory Receptor Family 51 subfamily E (OR51E) |
| --- | --- | --- | --- |
| FFAR3, FFA3R | GPR41 | - | - |
| FFAR2, FFA2R | GPR43 | - | - |
| - | GPR109a | HCAR2 |  |
| - | - | - | OR51E2 (Olfr78 in mice) |
| - | GPR136, GPR164 | - | OR51E1 |
| FFAR3L | GPR42 | - | - |
| FFAR1, FFA1R | GPR40 | - | - |

Supplemental Table 2

| Receptor | Ligand(s) | Tissue mRNA Expression (nTPM)^a^ | Protein Expression^b^ | Source |
| --- | --- | --- | --- | --- |
| OR51E2 (PSGR) [Olfr78 in mice] | Acetate = Propionate | Prostate (28.0)  Colon (9.3)  Placenta (3.8)  Seminal vesicle (2.2)  Rectum (2.1)  Smooth muscle (2.0)  Endometrium (1.2) | Data not provided | (58) |
| OR51E1 (GPR136, GPR164, OR51E1P, OR52A3P) |  | Placenta (7.2)  Prostate (6.4)  Adipose tissue (5.5)  Heart muscle (4.5)  Tongue (3.8)  Ovary (2.7)  Breast (2.3)  Skeletal muscle (2.0)  Colon (1.7)  Small intestine (1.2)  Smooth muscle (1.0) | Lung^H^  Salivary gland^H^  Rectum^H^  Testis^H^  Appendix^H^  Thyroid gland^M^  Nasopharynx^M^  Oral mucosa^M^  Gallbladder^M^  Urinary bladder^M^  Prostate^M^  Vagina^M^  Endometrium^M^  Cervix^M^  Skin^M^  Tonsil^M^  Bone marrow^M^  Duodenum^L^  Colon^L^  Small intestine^L^ | (180) |
| GPR42 (FFAR3L, GPR41L, GPR42P) | Long-chain fatty acids (>12 carbons) | Adipose tissue (0.5)  Colon (0.3)  Breast (0.3) | Data not provided | (181) |
| GPR40 (FFA1R, FFAR1) | Medium-chain fatty acids (6-12 carbons) | Bone marrow (3.0)  Pancreas (2.4)  Ovary (2.4)  Spinal cord (2.3)  Midbrain (1.1)  Hippocampal formation (1.1) | Data not provided | (182) |
|  |  | Basophil (1.0) |  |  |
| FFAR3 (FFA3R, GPR41) | Butyrate = Propionate > Acetate | Adipose tissue (3.9)  Appendix (2.4)  Breast (2.2)  Smooth muscle (1.5)  Colon (1.2)  Spleen (1.2)  Choroid plexus (1.0) | Duodenum^L^  Colon^L^  Small intestine^L^  Rectum^L^ | (183) |
|  |  | Neutrophil (2.8)  Eosinophil (2.5) |  |  |
| FFAR2 (FFA2R, GPR43) | Acetate = Propionate > Butyrate | Spleen (21.7)  Bone marrow (20.0)  Appendix (17.3)  Lung (7.3)  Urinary bladder (5.8)  Adipose tissue (4.7)  Small intestine (4.5)  Tongue (3.5)  Duodenum (3.2)  Breast (2.2)  Liver (1.5)  Placenta (1.5)  Gallbladder (1.4)  Esophagus (1.3)  Smooth muscle (1.3)  Stomach (1.2)  Prostate (1.0)  Fallopian tube (1.0)  Skin (1.0) | Spleen^H^  Caudate^M^  Lung^M^  Bone marrow^M^  Duohumadenum^n/d^  Colon^n/d^  Small intestine^n/d^  Rectum^n/d^ | (184) |
|  |  | Neutrophil (249.8)  Eosinophil (85.0)  Non-classical monocyte (6.5)  Intermediate monocyte (5.6)  Basophil (3.1)  Classical monocyte (2.1) |  |  |
| GPR109a (HCAR2, HCA2, HM74A, NIACR1, Puma-g, PUMAG) | Butyrate | Esophagus (39.7)  Skin (29.3)  Spleen (25.3)  Bone marrow (21.9)  Adipose tissue (21.4)  Seminal vesicle (14.2)  Salivary gland (13.7)  Vagina (13.6)  Lung (11.5)  Thymus (10.2)  Tonsil (9.9)  Appendix (9.6)  Cervix (8.7)  Breast (7.3)  Urinary bladder (6.7)  Parathyroid gland (5.3)  Placenta (4.4)  Kidney (3.8)  Prostate (3.3)  Liver (2.8)  Testis (2.7)  Tongue (1.9)  Stomach (1.5)  Small intestine (1.0) | Urinary bladder^M^  Seminal vesicle^M^  Spleen^M^  Lymph node^M^  Tonsil^M^  Bone marrow^M^  Duodenum^L^  Colon^L^  Small intestine^n/d^  Rectum^n/d^ | (49) |
|  |  | Neutrophil (177.3)  Basophil (38.8)  Non-classical monocyte (24.0)  Intermediate monocyte (5.3)  Eosinophil (2.6)  Classical monocyte (2.5)  Myeloid DC (1.7)  Total PBMC (1.4) |  |  |

*Tissue Expression based on data from cited sources as well as (50, 51. ^a^Only tissues with normal transcription per million (nTPM) ≥ 1.0, or the top 3 tissues with the highest expression are shown in this table receptor ^b^Protein expression classified as high, medium, or low. Selected information presented as relevant to the discussion of this review. High expression denoted by superscript “H,” medium expression denoted by superscript “M,” low protein expression denoted by superscript “L,” and protein expression that is not detectable is represented by superscript “n/d.” GPR: G-protein receptor; FFAR: Free fatty acid receptor; HCAR: Hydroxycarboxylic acid; PBMC: Peripheral blood mononuclear cells, which includes T-lymphocytes, B-lymphocytes, natural killer (NK) cells, monocytes, and dendritic cells; DC: Dendritic cells. Monocyte subsets (52) include classical monocytes: can differentiate into macrophages in tissues and can contribute to chronic disease [CD14^+^ CD16^–^], non-classical monocytes [CD14^–^ CD16^+^], and intermediate monocytes [CD14^+^ CD16^+^] which are discussed further in the section “SCFAs and the immune system.”*

Supplemental table 3: Antibiotic classifications


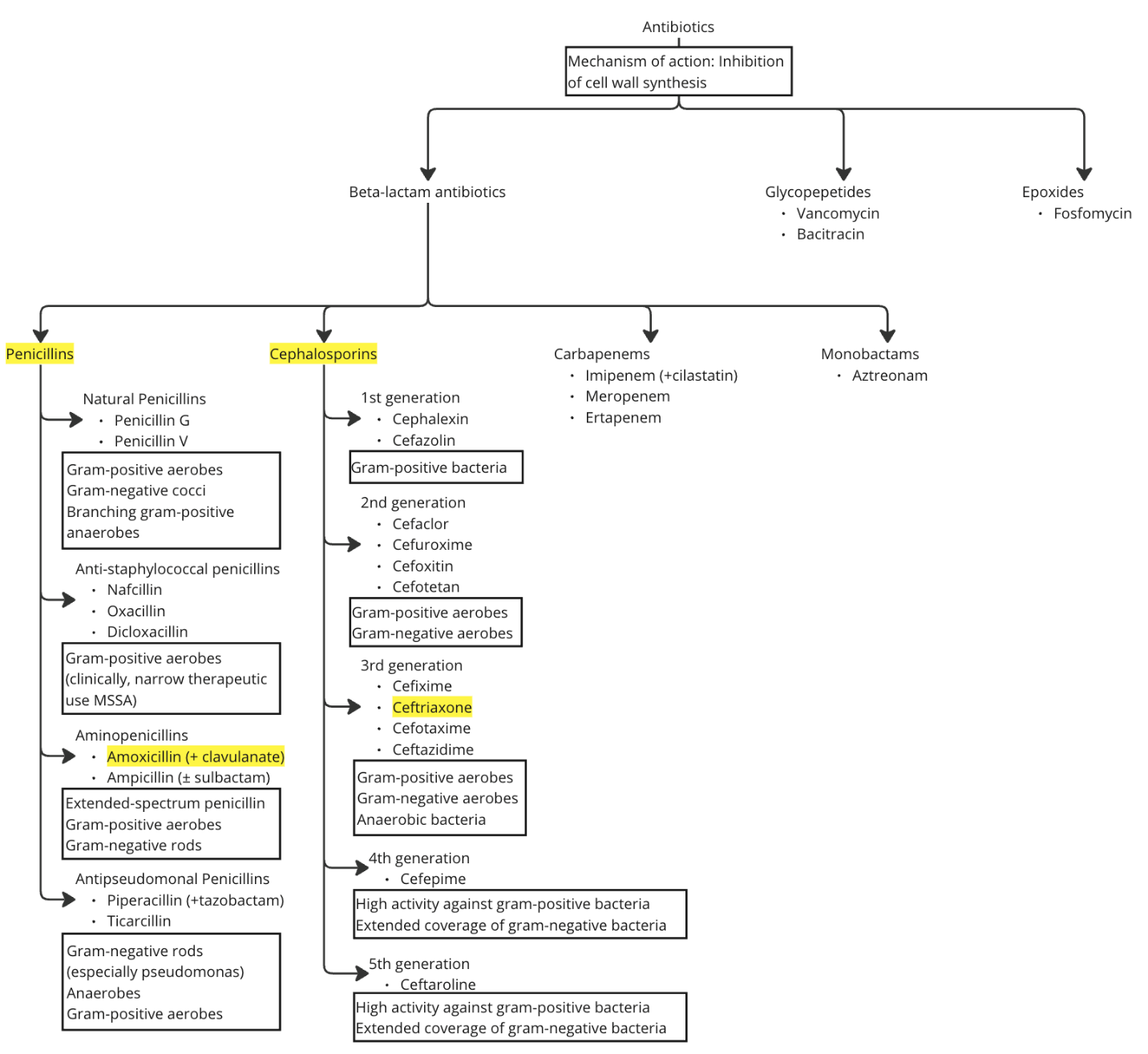


Visual breakdown of the antibiotics that act via inhibition of cell wall synthesis. Additionally, shows the breakdown of beta-lactam antibiotics and the bacteria they have proven efficacy in treating. Sources for diagram (185, 186).

Supplemental Information Sources

^180^N.A. OR51E1. The Human Protein Atlas. https://www.proteinatlas.org/ENSG00000180785-OR51E1.

^181^N.A. GPR42. The Human Protein Atlas. https://www.proteinatlas.org/ENSG00000126251-GPR42.

^182^FFAR1. The Human Protein Atlas. https://www.proteinatlas.org/ENSG00000126266-FFAR1/tissue.

^183^FFAR3. The Human Protein Atlas. https://www.proteinatlas.org/ENSG00000185897-FFAR3.

^184^FFAR2. The Human Protein Atlas. https://www.proteinatlas.org/ENSG00000126262-FFAR2/tissue.

^185^N.A. Ceftriaxone. Drugbank Online. https://go.drugbank.com/drugs/DB01212 (2024).

^186^N.A. Overview of antibiotic therapy. AMBOSS. https://next.amboss.com/us/article/mm0VTg?q=antibiotics) (2024).
